# A proposed production method for astatinated (At-211) Trastuzumab for use in a Phase I clinical trial

**DOI:** 10.1371/journal.pone.0307543

**Published:** 2024-09-24

**Authors:** Emma Aneheim, Tom Bäck, Holger Jensen, Stig Palm, Sture Lindegren

**Affiliations:** 1 Department of Medical Radiation Sciences, Institute of Clinical Sciences, Sahlgrenska Academy, University of Gothenburg, Gothenburg, Sweden; 2 Department of Oncology, Region Västra Götaland, Sahlgrenska University Hospital, Gothenburg, Sweden; 3 Department of Clinical Physiology and Nuclear Medicine, Cyclotron and Radiochemistry unit, Copenhagen University Hospital, Copenhagen, Denmark; University of Utah, UNITED STATES OF AMERICA

## Abstract

Astatine-211 is a nuclide with a short half-life of 7.2 h, that show promise for targeted alpha therapy of disseminated cancer. Despite nuclide production being straight-forward using a medium energy cyclotron and an uncomplicated target, not many cyclotrons are currently producing the nuclide. In this work we propose a stream-lined method to produce astatine labelled antibodies that enable production of clinical doses at other sites, remote from the nuclide producing cyclotron. Preconjugating the antibody prior to labelling, quick and efficient astatine recovery from the irradiated target in combination with optimized nuclide production logistics and an efficient synthesis for labelling are all key components to produce a clinical amount, > 300 MBq, of astatinated Trastuzumab.

## Introduction

Targeted alpha therapy is an emerging technique utilizing the high linear energy transfer and destructive power of the alpha particle directed to cancer cells by an organic or biological targeting vector. Alpha particles have a short range in tissues (<100 μm) and by depositing high amounts of energy (> 4 MeV) this results in a high probability for DNA double strand breaks and death of targeted cells [1].

When comparing the α-emitting radionuclides proposed for nuclear medicine applications, astatine-211 emerges as one of the most promising nuclides for successful and potentially curative treatments [2]. Astatine-211 has a 7.2 h half-life and can be produced comparatively cost-effective in useful yields, up to 9 GBq, with a medium energy cyclotron. The target raw material for this reaction, ^209^Bi (α, 2n)^211^At, is isotopically pure natural bismuth, which makes target production and handling simple, safe and cheap [3]. Astatine-211 decay with 100% alpha particle emission, one alpha per decay, and have no long-lived toxic daughter nuclides. This allows for more accurate dose calculations, in contrast to serially decaying nuclides with alpha-emitting daughters and motivates use in up-front treatments. In the decay of astatine-211, only a very small fraction of high energy gamma photons is emitted, which makes radiation protection comparatively easy. The characteristic X-rays of 75–93 keV emitted during the decay allow for both easy detection of astatine-211 as well as gamma camera imaging.

Many preclinical studies with astatine-211 include tumour specific antibodies as vectors. Antibodies do, however, normally have a long circulation time in vivo, > 48 h, and are therefore not compatible with the short half-life of astatine-211 in systemic applications. Two previous clinical studies have been performed with astatine-211 labelled antibodies and antibody fragments. Both, however, for intracavitary applications; one on malignant glioma in Durham, USA and one on ovarian cancer in Gothenburg, Sweden [4–6]. Both these studies were completed more than 10 years ago. Since then, the regulatory landscape around clinical trials, and especially radiopharmaceutical production for clinical trials, has changed significantly, with the introduction of new regulations and guidelines.

In this work we present a proposed clinical GMP production method and quality control scheme, for the tumour specific antibody trastuzumab labelled with astatine-211. The primary intended use is in an updated Phase I clinical study for intraperitoneal treatment of disseminated ovarian cancer to be conducted at the Sahlgrenska University hospital in Gothenburg, Sweden.

In early-stage ovarian cancer the patients usually present few symptoms and clinical signs, and it is common that intraperitoneal dissemination has already occurred at the time of diagnosis. Therefore, more than half of these patients will experience a relapse within two years, despite standard treatment by surgery and chemotherapy. This relapse mainly occurs in the abdominal cavity, which is why novel therapies, such as targeted alpha therapy with astatine-211 labelled antibodies, which has high potential to prevent intra-abdominal relapse, are desired [7, 8]. Since free astatine-211 leave the peritoneal cavity quickly, labelling it to an antibody serves both to retain the nuclide within the peritoneum for a longer time, and also to facilitate specific binding to the cancer cells.

The intended clinical application is an intraperitoneally administered osmotic solution of large volume (1–1,5 L) in which the astatine-211 labelled antibody is infused at a desired activity concentration with high specific activity. Dosimetry and biokinetic modeling have shown that for intraperitoneal targeted alpha therapy of ovarian cancer microtumors, the main tumor dose originates from decay of cell-bound astatine-211 radioactivity [9]. Therefore, an increase in the specific activity would further increase the tumor dose without affecting the dose to normal organs, including the bone marrow. After ca 24 h, when the main part of the activity has decayed, the solution is removed from the abdominal cavity. This radiopharmaceutical should preferably be administered as an adjuvant single-step treatment after primary debulking surgery in order to prevent relapse.

Previous dose calculations and preclinical experiments show that a total administered activity of 300 MBq (200 MBq*L^-1^) of the astatine-211-antibody into the peritoneal cavity results in absorbed doses that are sufficient for eradication of single tumour cells and small micrometastes in this treatment modality [9]. *In vivo* stability and biodistribution of astatinated Trastuzumab has previously been assessed in mice [10]. This is, however, not directly translatable to the human case as leakage from the peritoneal cavity largely influence the stability due to intracellular metabolism and this process is more rapid in the mouse than in humans. Therefore, the previous clinical study performed in Gothenburg gives a better indication of the in-vivo stability of astatinated antibodies. Data from the prior Phase I study show that the level of administered activity was safe with no observed radiation related toxicity [5, 6].

In the current work we describe the development of an efficient and robust GMP production method for clinical use of astatine-211 labeled antibodies. Even though the method for producing astatine-211 is well established, a medium energy cyclotron is needed and there are few facilities world-wide producing this radionuclide [3]. In Europe, only two cyclotrons currently produce astatine-211 on a regular basis and the closest cyclotron to the clinical site in Gothenburg, Sweden, is at Copenhagen University Hospital in Denmark. The proposed radiopharmaceutical production described in this work will therefore be performed in a node-like fashion, in close collaboration between the clinical and pharmaceutical production site in Gothenburg, Sweden and the nuclide production facility in Copenhagen, Denmark.

## Results

### Logistics of nuclide production and recovery

Cyclotron irradiation is performed during the late evening/night and for a 4 h beamtime result in activities between 1.2–2.8 GBq at the end of bombardment, EOB. After 30 minutes cooling, the At-211 activity and presence of any contaminants is measured using a High Purity Germanium (HPGe) detector. Everything is documented, including the different transport labels of the containers. Astatine-210 is an often-mentioned unwanted side product in the production of astatine-211 due to its decay to polonium-210. With a 29 MeV α-beam the co-production of astatine-210 amount to approximately 0.012% of the astatine-211 activity. The total activity values for exemption of polonium-210 is 10 kBq, which is not relevant below 0.4% co-production of astatine-210 at an astatine-211 activity of 1 GBq [11]. It is therefore not considered a radiation safety problem.

**Fig 1:**
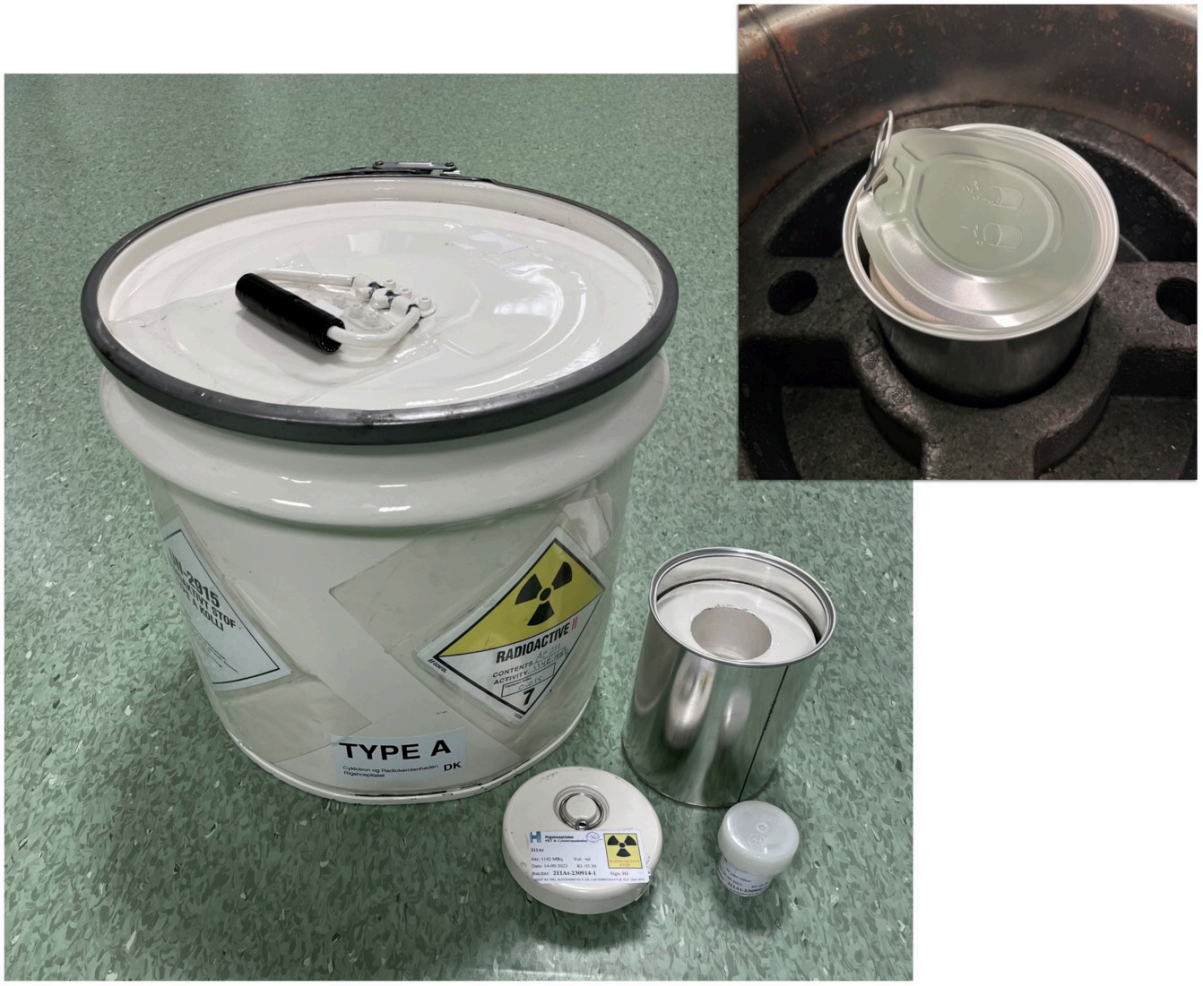
Illustration of all components of the TYPE A approved colli used for transport of the irradiated astatine-211 target.

The irradiated target is packaged from the inside out in: 1. a small plastic screw top can, 2. a small lead container, 3. a sealed tin can which in turn is placed in an outer metal barrel, Fig 1. This construction has been approved as a TYPE A colli for dangerous radioactive goods by the Danish authorities according to ADR 2021. The target is then transported by road to Gothenburg, Sweden, crossing country borders, which takes approximately 3.5 h. Taken together, this results in an actual target activity upon arrival in Gothenburg of 60 ± 3% (n = 18, ST1 Table in S1 File) of the activity at release after packaging, 1 h after EOB, based on astatine deliveries during a time-period of 5 months. With a high current irradiation, target activities at arrival in Gothenburg are > 1,5 GBq (ST1 Table in S1 File).

After target arrival in Gothenburg, the automatic astatine recovery process through dry distillation follows. Based on 21 recovery processes, also during a time-period of 5 months, it takes *circa* 30 minutes from starting the dry distillation program to collecting the purified astatine solvated in chloroform and measuring the activity in a dose calibrator. The first step of the process is preconditioning of the system, with respect to temperatures, and this is typically finished within 12 minutes. The resulting average yield of the recovery process is 84 ± 7%, decay corrected (n = 23, ST2 Table in S1 File). After collecting the purified astatine in chloroform, the chloroform solution is evaporated to dryness in the automatic equipment to achieve a dry activity residue. This step takes *circa* 10 minutes, depending on the eluted volume, and the retention of activity is 99.4 ± 1.1% (n = 8, ST3 Table in S1 File). For clinical preparation the dry astatine residue is transferred to a Class C preparation room for radiolabelling under GMP conditions. The reason for separating these two steps will be presented in the next section.

### Defining the production process

As partly described above, several different process steps will be involved in the future production and formulation of the final astatine-211-radiophamaceutical infusion product i.e. the osmotic liquid with the astatinated Trastuzumab to be used for intraperitoneal patient administration. The process steps include: 1. Target production, 2. Antibody conjugate prefabrication, 3. Target irradiation, 4. Astatine recovery from target, 5. Radiolabeling, 6. Sterile filtration and 7. Infusion of astatinated antibody in the osmotic liquid.

**Fig 2:**
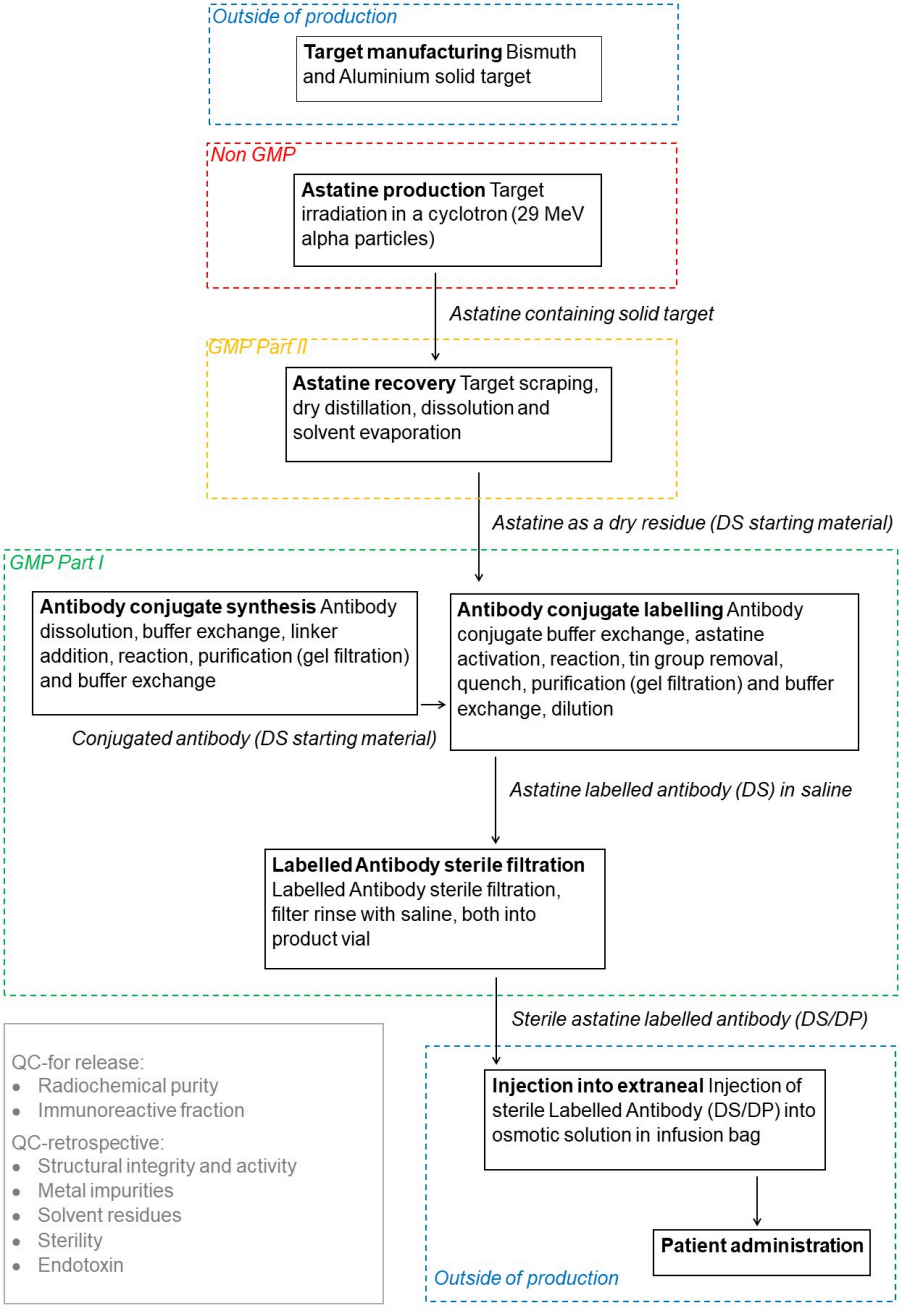
Schematic description of the production process from cold target to astatine labelled antibodies dissolved in an osmotic infusion liquid.

Not all these steps are necessary to include in the actual drug product GMP production. This is illustrated in Fig 2 below, which also details the entire production process. Radionuclide production is normally not GMP classified and hence the target preparation also falls outside of the production process. In the proposed production process also infusion of the astatinated antibody into the osmotic solution will be excluded from GMP production, instead being defined as preparation. This excluded the need for advanced quality control of the osmotic fluid, which is already an approved medicinal product. Perhaps less obvious is that the recovery of astatine from the target is classified as GMP part II rather than part I. This was necessary in order to be able to utilize non-classified facilities for the astatine recovery process, which result in the dry astatine residue being classified as a drug substance starting product.

In this case the proposed production process is classified as radiopharmaceutical production and hence the facility where it is undertaken needs a manufacturers licence that allow production for clinical trials. The manufacturers licence requires qualification of a wide range of involved equipment and significant administrative work that can be minimized by keeping the complexity of the process as low as possible. With the proposed process described here, a licence for the production of astatinated Trastuzumab for clinical trials was obtained at the Nuclear Medicine production site in Gothenburg.

### Radiopharmaceutical production

The immunoconjugate, Trastuzumab linked to the bispecific reagent N-succinimidyl-3-trimethylstannyl-benzoate (m-MeATE), is prefabricated in-house the day before astatine delivery and radiolabelling in a Class C preparation room under GMP conditions. The conjugate is stored until use under physiological conditions in phosphate buffered saline, PBS pH 7.4, at 4°C.

As the dry astatine recovered from the automatic equipment is delivered in a comparatively large vial (4 mL), the preclinical manual labelling protocol must be adapted and scaled to fit these conditions for clinical preparation. To efficiently oxidize the astatine and cover the bottom of the 4 mL vial, a volume of at least 40 μL of Methanol with 1% acetic acid and the oxidizing agent N-iodo succinimide must be used. Not to negatively affect the antibody, an immunoconjugate volume of > 450 μL then have to be applied, to keep the methanol concentration in the resulting solution < 10%. The large volumes in turn mean that a low antibody concentration, dilutions of either 0.4 or 0.2 mg*mL^-1^, have to be employed in order to achieve a high specific activity product, aiming at 1 GBq*mg^-1^. To achieve a high radiochemical yield with these demanding conditions, the reaction buffer was found to be very important, in which 0.2 M Acetate buffer of pH 5.5 rendered the best radiochemical yields of 75 ± 4% (n = 10, ST4 Table in S1 File).

The clinical radiolabelling protocol furthermore have to be based on the ingoing dry residue of astatine activity from the automatic recovery process, in order to achieve the desired high specific activity product without compromising yields and product quality. This means varying volumes and concentrations of the prefabricated antibody conjugate and N-iodo succinimide quench solution at every production occasion. Utilizing the simple scheme defined in Table 1, very high specific activities are possible to achieve consistently while maintaining the high radiochemical yield of the reaction, SA = 1.23 ± 0.1 GBq*mg^-1^ (n = 10, ST4 Table in S1 File).

**Table 1:** Calculation scheme for synthesis of astatinated Trastuzumab with a specific activity of 1 GBq*mg^-1^, starting from the available amount of dry astatine-211 activity.

Measure the astatine activity A in MBq.	
Is A < 300 MBq?	If YES use c(conjugate) = 0.2 mg*mL^-1^,
	If NO use c(conjugate) = 0.4 mg*mL^-1^.
Calculate the mass conjugate, m, needed	m = (A*0.6)/(1000)^-1^ (mg)
Calculate the conjugate volume, V	V = m/c (mL)
Is V> 0.45 mL?	If YES V = V
	If NO V = 0.45 mL
Calculate the volume of NIS quench solution V_N_	V_N_ = (m*10) +1 (μL)

To purify the astatinated Trastuzumab from presence of non-reacted “free” astatine and other small molecular compounds such as oxidizing agent, solvents, substituted tin groups et.c. gel filtration, or size exclusion, is applied. The prepacked PD10 Column from Cytiva is proposed to be used without modification, relying on sterile filtration of key solutions and buffers, including, of course, the final product. Sterile filtration of the astatinated antibody solution after gel filtration introduce accumulated activity/product losses of 15 ± 3% (n = 8, ST5 Table in S1 File), despite using low protein binding sterile filters. The final volume of the product after sterile filtration is diluted to a volume of 15 mL to reduce dose and maintain stability as well as to allow for a sufficient volume for quality control.

### Quality control

Presence of free, i.e. non-antibody bound, astatine is assessed through determination of the radiochemical purity via methanol precipitation of the protein. This is a method developed specifically for astatine labelled proteins that has been in use for > 20 years [12]. This quick assay takes less than 20 minutes and shows a radiochemical purity of the product produced according to the previously described protocol of 96 ± 2% (n = 10, ST6 och ST7 Tables in S1 File). The product is stable in terms of maintained radiochemical purity at room temperature for > 24 h (ST7 Table in S1 File), although it will never be relevant to store the product for more than one half-life i.e. circa 7 hours, before patient administration.

Maintained immunoreactive properties of the radiolabelled Trastuzumab is assessed through binding to HER2-coated magnetic beads. This assay was developed for drug product release and gives an immunoreactive fraction, expressed in terms of Bound antibody over Total added antibody (B/T), of 0.78 ± 0.02 (n = 5, ST8 Table in S1 File) after 20 minutes. When comparing this immunoreactive fraction to that achieved through binding to different batches of HER2 expressing SKOV3 cells, a similar result was observed with a B/T = 0.76 ± 0.02 (n = 6, ST9 Table in S1 File) after 3 hours. Both these results can be compared to the immunoreactivity of directly iodinated unconjugated Trastuzumab, serving as a reference compound, which gives a B/T = 0.75 ± 0.02 (n = 5, ST10 Table in S1 File) with the magnetic beads method. This shows and subsequent astatination. Binding of an unspecific antibody using the assay gives B/T = 0.04 ± 0.02 (n = 3, ST9 Table in S1 File), showing a high specificity of the method. It is, however, important to only use freshly dissolved beads as performance decrease significantly upon storage of the beads after opening.

An in-house FPLC analysis will also be performed retrospectively in order to make sure that the antibody maintain its structural integrity i.e. that it has not deteriorated into fractions or formed aggregates during the chemical processing. This is assured by comparing the retention time of the activity of the astatinated Trastuzumab by fraction collection to that of the online UV absorption of the non-labeled antibody, making sure the deviation is less than ± 1 minute, S1 Fig, ST11 and ST12 Tables in S1 File. Repeated FPLC analysis after > 24 h storage of the product at room temperature show that the main part of the radioactivity still is associated with an intact antibody, S1 Fig, ST11 Table in S1 File.

The validation procedure of the in-house QC methods of methanol precipitation, binding to HER2 magnetic beads as well as FPLC analysis according to repeatability, robustness and specificity is described in the S1 File.

Due to the chemical processing of the radionuclide and antibody described in the methods section, there is a risk of presence of the residual solvents chloroform and methanol as well as trace metals aluminium, bismuth and tin. This warrants for retrospective analyses by GC-MS and ICP-MS by contract laboratories offering qualified and validated analytical methods. Analysing test batches show that with the proposed production method, presence of all proposed contaminants is well below the predetermined safety standards according to the European Pharmacopeia section 2.4 [13].

Besides standard filter integrity testing (bubble point test) of the sterile filter before product release, remains in the product vial will be analysed for sterility and pyrogens by contract laboratories using direct inoculation and endotoxin analysis by Limulus Ambeocyte Lysate (LAL) test according to the European Pharmacopeia section 2.6 [13].

## Discussion

The work with developing the presented clinical production method for astatine-211 labeled Trastuzumab is a process that has been ongoing for several years, where different parts, such as logistics, production process and chemistry has been developed both in parallel and in sequence. The methods were adopted to meet all the requirements for clinical use of astatinated antibodies.

In Gothenburg, Sweden, there are no cyclotrons capable of producing astatine-211. The closest cyclotron capable of producing an alpha beam for the production of astatine-211 is located in Copenhagen, Denmark, at a distance of 269 or 316 km by road, depending on the means to cross the in/outlet of the Baltic Sea, by ferry or bridge, respectively. To achieve sufficient clinical amounts of activity of the astatine-211 radiopharmaceutical in Gothenburg, the production logistics have to be streamlined, including irradiation, cooling and QC at the cyclotron, followed by transport to Gothenburg. On the same note, also nuclide recovery and the subsequent radiopharmaceutical synthesis and product QC must be optimized.

The already efficient logistical process described herein can be further optimized by also aligning nuclide recovery with the target delivery, as the target does not need to be inserted into the dry distillation equipment until after the preconditioning step, reducing the total time even further.

Another important step for streamlining the production process was achieved when it was concluded that the antibody conjugate could be manufactured prior to the astatination without compromising the quality of the labelled product [14]. This means that the conjugate will be prefabricated the day before radiolabelling and patient treatment. In this way, radiolabelling can start immediately after the astatine has been recovered from the target; a process which in this case is significantly faster than preparing an antibody immunoconjugate.

**Fig 3:**
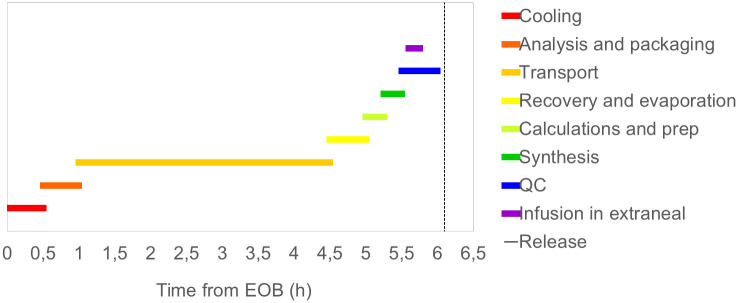
Overview of the optimized time consumption of the different steps involved in the production of astatinated Trastuzumab.

Also keeping quality control for release of the product at a minimum, with parallel assays that can be conducted in less than half an hour, as described above, a proposed total time for production of less than one half-life of astatine-211 starting from EOB can be achieved, as illustrated in Fig 3.

Besides pure logistics, a high non decay corrected radiochemical yield of the labelling reaction is important to ensure that enough activity will be available to perform the planned patient treatment. This is especially true as a significant product/activity loss is introduced in the inevitable sterile filtration step of the final product.

Sterility is one of several analysis that cannot be made prior to batch release but will be made retrospectively to make sure that the product is safe and intact. Because of this retrospective type procedure, analysis of validation batches prior to starting the clinical trial are important in order to make sure that the proposed production method meet all the set standards.

When performing clinical trials with a rather short-lived nuclide such as astatine-211 only a few short and efficient quality control assays can be performed before release of the drug product. In the proposed method, presence of free, i.e. non-antibody bound, astatine and maintained antigen binding of the antibody have been deemed as the most important parameters to assess prior to patient release. As a full cell assay for determination of Immunoreactive fraction of the astatinated antibody, according to Lindmo et al. [15] takes too long to be used as release criteria an alternative method was developed using HER2-coated magnetic beads to test binding of the radiolabelled Trastuzumab. Although the developed labelling method is very robust, preclinical experience give that if the labelling yield would result in low radiochemical yield, < circa 30–40%. This will compromise specific activity and likely affect radiochemical purity, leading to a product which cannot be released. This highlights the importance of the quality control method in which presence of free astatine in the product can be detected.

The production method proposed in this work is a hybrid between automation and manual work in that the recovery of astatine from the irradiated target and subsequent solvent evaporation is conducted with an automatic equipment while the radiopharmaceutical synthesis is performed manually, utilizing the dry astatine as a starting material. The automatic recovery method, that has never been employed in a clinical setting before, allow for a very robust and reliable astatine recovery yield with significantly increased radiation safety for the personnel compared to previous manual methods used [4, 5]. This proposed method will make it possible to efficiently produce astatinated antibodies, Trastuzumab, of GMP quality for clinical trials in a production facility located circa 300 km from the nuclide producing cyclotron, crossing country borders. The labelling protocol developed in this work allow for consistent specific activities that are an order of magnitude higher than previously reported GMP production of astatinated antibodies [16]. Streamlining the process all the way from target irradiation to radiopharmaceutical synthesis as described above will ensure the possibility to produce patient batches with activities > 300 MBq. This method will hence pave the way for astatine-211 based treatments in a node-type fashion located around available production sites.

## Materials and methods

All uncertainties presented are calculated as standard deviations (1 σ).

### Astatine production & recovery

Astatine-211 is produced in a Scanditronix MC32 cyclotron located at Copenhagen University Hospital, Denmark, utilizing the nuclear reaction ^209^Bi (α, 2n)^211^At. The target consists of an aluminium backing coated with 24±2 mg*cm^-2^ bismuth covered with a layer of 0,3–0,6 mg*cm^-2^ of aluminium. The aluminium coating is aimed to reduce the diffusion of astatine from the target during irradiation. The target is irradiated with a beam energy of 29 MeV alpha particles. To avoid target melting the beam current is presently limited to 35 eμA. In a 4 hour irradiation typically 1.2–1.5 GBq of astatine-211 is produced at EOB applying 17 eμA, and 2.8–3 GBq EOB using 32 eμA. The unwanted astatine-210 is co-produced at this beam energy with an At-210/At-211 ratio of 1.2*10^−4^. Contaminants like the isotopes sodium-24, aluminium-29 and magnesium-27 are also co-produced on a level comparable to astatine-210 at EOB. HPGe gamma spectroscopy is used to analyse the irradiated target and measure the produced activity of astatine-211, astatine-210 and contaminants. A 15% GC1520-7500SL coaxial high purity germanium detector from Canberra Industries is used. Calibrations of the detector are performed with traceable NIST sources ^133^Ba and ^152^Eu sources (AEA Technology QSA GmbH). Spectra are analyzed using the Genie 2000 software from Canberra.

After transport, astatine-211 is recovered from the solid aluminium target using dry distillation with the Atley C100, Atley Solutions AB, similarly to a process previously described [17]. Separated astatine is eluted from the cold trap using 500 μL of Chloroform. The chloroform is then evaporated in the same equipment using a gentle nitrogen flow of *circa* 100 mL*minute^-1^, rendering a dry residue of astatine in a 4 mL silanized screw cap glass vial.

### Antibody conjugation

The antibody conjugate is formed by reacting Trastuzumab with the bispecific reagent N-succinimidyl-3-trimethylstannyl-benzoate (m-MeATE) that allow for subsequent astatine labelling. Trastuzumab is obtained as Herceptin^®^ 150 mg and dissolved in sterile water according to instructions. The resulting antibody solution of 21 mg*mL^-1^ is buffer exchanged twice using prepacked NAP10 size exclusion columns from Cytiva to a final concentration of 4 mg*mL^-1^ in 50 mM borate buffer, pH 8,5. From a stock solution of 50 mg*mL^-1^ of m-MeATE in chloroform, 2 μL is evaporated to dryness and redissolved in 15 μL DMSO. 7,5 μL of the m-MeATE solution is added to 0,75 mL, 3 mg, Trastuzumab solution, rendering a molar excess of 7 m-MeATE per antibody. The reaction mixture is incubated for 30–45 minutes on a gentle vortex before being purified on a NAP10 column and diluted to a final concentration of 1 mg*mL^-1^ in PBS, pH 7,4. This reaction likely results in a Poisson distributed conjugation between 0–7 m-MeATE per antibody with an average between 3–4 [18]. The final antibody conjugate solution is stored at 4°C until use.

### Radiolabelling

Two solutions of N-iodo succinimide (NIS) in 1% Acetic acid in methanol is prepared; NIS-1 = 44μM, NIS-2 = 4,4 mM. Before radiolabelling the prefabricated antibody conjugate between Trastuzumab and m-MeATE is buffer exchanged to 0,2 M Acetate buffer pH 5,5 to a final concentration of 0,4 mg*mL^-1^. The activity of the dry astatine vial is measured using a dose calibrator and if the activity is below 300 MBq the antibody solution is diluted to 0,2 mg*mL^-1^ in order to achieve the desired specific activity. To the dry astatine vial, 40 μL NIS-1 solution is added and incubated on gentle vortex for 30 seconds. The desired volume of antibody conjugate and NIS-2 solution are calculated according to Table 1 and subsequently added to the reaction vial and the reaction allowed to proceed for 1 minute on gentle vortex respectively. 5 μL ascorbic acid, 50 mg*mL^-1^ in water, is added to the reaction vial and the mixture incubated for 30 seconds. The astatinated Trastuzumab is then purified using a PD10 column from Cytiva and eluted and diluted in sterile 0,9 mg*mL^-1^ NaCl solution for injection from B. Braun to a final volume of 10 mL. The activity is measured again in order to calculate the radiochemical yield of the reaction. The product solution is then sterile filtered into an evacuated 25 mL sterile product vial from ABX using a 0,22 μm low protein binding filter and diluted to a final volume of 15 mL.

### Quality control

#### Methanol precipitation

Methanol precipitation precipitates the radiolabeled antibody while leaving free astatine-211 in solution. In this way the radiochemical purity can be calculated. Circa 5–20 kBq of the astatinated Trastuzumab is added in triplicates to 200 μL 1% Bovine Serum Albumin in PBS. After homogenization 500 μL of methanol is added to the samples whereby the protein mixture precipitates. The samples are centrifuged for 10 minutes after which the supernatant is removed and the protein pellet is measured for activity. The radiochemical purity is then calculated as % of activity in the pellet compared to the total activity added to the sample in form of triplicate reference samples.

#### Binding assays

To assess maintained binding of the astatinated Trastuzumab to its tumor target, a beads assay was developed. Magnetic beads, 2 μm particle size, substituted with 43 μg of the human epidermal growth factor 2 (HER2) per mg of beads, resulting in a binding capacity of > 27 μg antibody per mg of beads are used (ACRO biosystems). The beads are reconstituted with water according to instructions rendering a final bead concentration of 1 mg*mL^-1^. The solution is magnetized in a 1,5 mL conical vial, forming a pellet of magnetic beads, and washed twice with 0,05% Tween in PBS, pH 7,4, by removing the supernatant followed by redissolution of the beads. The beads are then diluted to a final concentration of 0,25 mg* mL^-1^ using the same buffer. 5 μL of the labelled antibody, diluted to 1 ng*μL^-1^, is added to 250 μL beads solution, in triplicates, which render a *circa* 300 times binding excess of the beads compared to the antibody. The solution is incubated for 20 minutes on gentle vortex. The beads are then magnetized and washed with 250 μL PBS-Tween before being redissolved and measured using a NaI(Tl) detector. The binding is assessed as B/T by comparing the activity of the beads (B) with the total activity added to the samples (T) in form of triplicate reference samples. The unspecific antibody Rituximab was used to assess unspecific binding to the beads in the above-described assay. Similarly, directly iodinated Trastuzumab was subjected to the above-described assay to act as a reference of unmodified antibody. In both these two cases, the antibodies were iodinated with iodine-125 using the mild Thermo Scientific Pierce Iodination Reagent, formerly known as Iodo-GEN, method [19].

The immunoreactivity of astatinated Trastuzumab was for comparison also assessed through binding to HER2 expressing SKOV3 cells (ATCC, Rockville, MD, USA). Six different cell batches of different age (between 4 and 20 years, stored in -100°C), were evaluated. The cell culture conditions were in all cases the same; 10% FBS (fetal bovine serum), 1% L-glutamine in McCoy’s media (Invitrogen, Life Technologies). The cultures were adherent, fed twice a week, and split once a week. In the cell assay, 5 μL of astatinated Trastuzumab, diluted to 1 ng*μL^-1^, was added to duplicate samples of cells, 0.5 mL, *circa* 5*10^6^ cells*mL^-1^, from each batch. The cell suspensions were then incubated in RT for 3 hours under gentle agitation before being centrifuged and washed with 1 mL of cold cell medium. The immunoreactivity was assessed as B/T by comparing the activity of the cell pellet (B) with the total activity added to the samples (T) in form of triplicate reference samples.

## Supporting information

S1 File Supporting information to the article: A proposed production method for astatinated (At-211) Trastuzumab for use in a Phase I clinical trial. (PDF)
