# Supplementary material for: A proposed production method for astatinated (At-211) Trastuzumab for use in a Phase I clinical trial
Source: PLoS One. 2024 Sep 24;19(9):e0307543. doi: 10.1371/journal.pone.0307543 (PMC11421776; doi:10.1371/journal.pone.0307543)
Supplement: S1 File — (PDF) [file pone.0307543.s001.pdf]

Supporting information to the article:

A Proposed Production Method for Astatinated (At-211) Trastuzumab for use in a Phase I Clinical Trial

Emma Aneheim [1,2]*, Tom Bäck [1] Holger Jensen [3], Stig Palm [1] and Sture Lindegren [1]

1. Department of Medical Radiation Sciences, Institute of Clinical Sciences, Sahlgrenska Academy, University of Gothenburg, SE41345, Gothenburg, Sweden.
2. Region Västra Götaland, Sahlgrenska University Hospital, Department of Oncology, SE41345, Gothenburg, Sweden
3. Department of Clinical Physiology and Nuclear Medicine, Cyclotron and Radiochemistry unit, Copenhagen University Hospital, 2100 Copenhagen, Denmark.

* *Correspondence: emma.aneheim@radfys.gu.se*

Table ST1. Target activity data produced during a 5 month time period and 17 eμA (white). Example of high beam current production using 32 eμA (grey).

A (Copenhagen release) MBq	Time	A (Gothenburg) MBq	Time	% of activity upon arrival
1449	03:30	850	08:17	59%
1410	03:30	894	07:42	63%
1439	03:30	859	07:44	60%
1531	03:30	944	07:46	62%
1404	03:30	814	08:05	58%
1394	03:30	788	08:38	57%
1509	03:30	924	07:43	61%
1103	03:30	666	07:48	60%
1400	03:30	893	07:23	64%
1082	03:30	643	07:42	59%
1470	03:30	860	08:20	59%
1219	03:30	708	08:00	58%
1047	03:30	563	08:45	54%
1230	03:30	780	07:30	63%
934	03:30	598	07:37	64%
1395	03:30	840	07:52	60%
1098	03:30	661	07:37	60%
1464	03:30	881	07:35	60%
2604	03:30	1565	07:37	60%
2726	03:30	1646	07:52	60%

Table ST2. Distillation data from a 5 month time-period (white). High activity distillations (grey).

A Target	Time	Time at program start	A Eluate	Time	Yield (DC)
666	07:48	08:10	476	08:45	0,78306
924	07:43	07:39	679	08:12	0,76980
788	08:38	08:39	586	09:12	0,78529
814	08:05	07:57	594	08:30	0,75955

944	07:46	07:48	638	08:22	0,71598
718	07:56	08:00	546	08:33	0,80689
859	07:44	08:08	636	08:45	0,81642
894	07:42	07:49	770	08:23	0,91978
850	08:17	08:07	744	08:44	0,91399
929	07:40	07:51	817	08:24	0,94368
693	07:43	07:50	590	08:24	0,90918
829	07:48	07:55	682	08:26	0,87432
713	08:30	08:24	570	08:56	0,83345
753	07:42	07:46	574	08:18	0,80755
727	07:41	07:51	609	08:23	0,89600
789	07:55	07:51	725	08:29	0,97033
890	07:38	08:04	662	08:40	0,82151
855	07:47	07:58	671	08:33	0,84482
749	07:45	07:48	602	08:34	0,86938
843	07:38	08:20	578	09:00	0,78192
715	08:00	08:28	535	09:02	0,82640
1565	07:37	07:55	1233	08:36	0,86597
1646	07:52	07:50	1145	08:32	0,74167

Table ST3. Automatic evaporation data.

A in (MBq)	time	A out (MBq)	time	Yield (DC)
208	10:44	199	11:04	0,98789
236	10:28	228	10:42	0,98802
274	12:26	265	12:47	0,98435
323	10:28	309	10:46	0,98465
346	10:25	345	10:36	1,01484
459	10:38	451	10:54	1,00809
672	08:56	656	09:07	0,99355
778	09:35	754	09:48	0,98955

Table ST4. Labelling and specific activity data

A in	A out	Yield (NDC)	m Mab (mg)	SA (GBq/mg)
134	96,3	0,7187	0,090	1,070
186	149	0,8011	0,120	1,242
197	153	0,7766	0,120	1,275
214	150	0,7009	0,133	1,131
219	163	0,7443	0,132	1,235
301	213	0,7076	0,183	1,164
319	235	0,7367	0,192	1,224
403	309	0,7667	0,240	1,288
509	369	0,7250	0,305	1,210
691	579	0,8379	0,400	1,448

Table ST5. Sterile filtration data

A in (MBq)	time	A out (MBq)	time	Yield (DC)
68,7	13:46	58,1	13:51	0,852508
94,2	12:54	74,8	13:00	0,801726
142	11:48	114	12:01	0,819715
153	11:20	127	11:27	0,839428
235	11:34	198	11:42	0,842553
309	12:16	274	12:22	0,895297
369	12:31	322	12:41	0,886723
559	11:15	497	11:20	0,896239

Table ST6. Radiochemical purity (RCP) data of labelled product, day of labelling

Before/Reference (CPM)	After (CPM)	RCP
42166	40198	
42748	40471	0,949828
43951	41728	
116815	111392	
118157	112391	0,952391
116897	*83359*	
149514	147198	
155087	154133	0,987389
148105	145707	
81822	81388	
85971	84239	0,985949
87334	85875	
163942	170324	
169442	174198	0,919928
155637	187064	
87637	78297	
84237	82221	0,942881
87038	83605	
228786	178925	
199815	204901	0,966855
182557	207076	
128767	117841	
110602	113394	0,991922
108877	119847	

Table ST7. Radiochemical purity (RCP) data of labelled product, one day after labelling

Before/Reference	After	

CPM	CPM	RCP
116406	135806	
127150	130832	0,944908
127893	126469	
400258	418651	
487096	461932	0,959045
508168	457786	

Table ST8. Binding to beads data, astatinated Trastuzumab

Before/ Reference =Total (CPM)	After = Bound (CPM)	Bound (B) /Total (T)
38242	28216	
46287	37878	0,805240
47000	38059	
20386	18129	0,755601
22218	18370	
84113	66122	
93025	73285	0,783118
87801	68086	
	66249	
89213	67671	0,751236
	67140	
64713	82694	
66514	84967	0,780221
63760	82253	

Table ST9. Binding to SKOV 3 cells, astatinated Trastuzumab

Before/ Reference =Total (CPM)	After = Bound (CPM)	Bound (B) /Total (T)
103189,81		
87434,98		
94996,42		
	75298,1	0,742668218
	66116,43	
	75077,01	0,791852538
	75702,91	
	74567,63	0,778969671
	73759,21	
	67953,13	0,703627997
	66027,59	
	70045,77	0,768527117
	76292,66	
	75233,82	0,805812215

	78204,22	

Table ST10. Binding to beads data, iodinated Trastuzumab and Rituximab

Antibody	Before/ Reference =Total (CPM)	After = Bound (CPM)	Bound (B) /Total (T)
	1406	999	
Trastuzumab	1389	970	0,713703
	1414	1035	
	1479	1083	
Trastuzumab	1260	1020	0,758469
	1496	1110	
	1289	931	
Trastuzumab	1264	928	0,756012
	1265	1028	
	2345	1730	
Trastuzumab	2380	1739	0,765006
	2247	1865	
		1712	
Trastuzumab	2313	1835	0,767376
		1777	
	1684,52	62,83	
Rituximab	1601,66	58,33	0,044363
	1660,01	98,27	
	1639,07	180,26	
Rituximab	1743,75	114,15	0,077629
	1559,43	89,25	
	1603,65	30,56	
Rituximab	1505,94	51,53	0,038228
	1696,04	101,62	

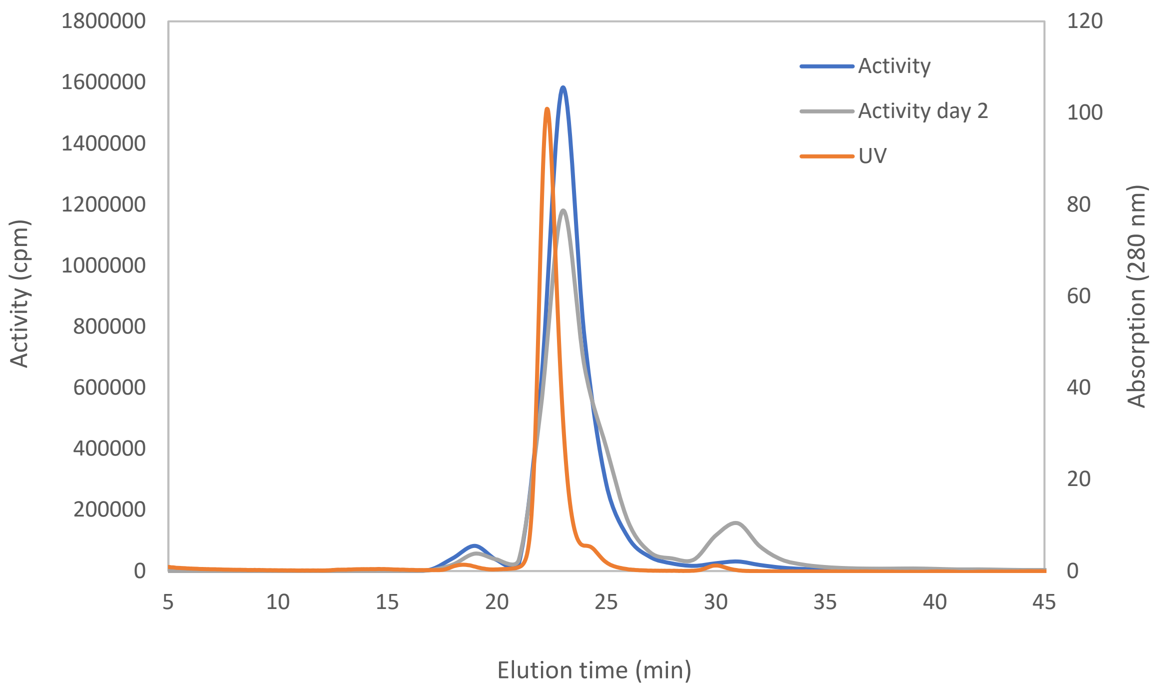

Figure S1. FPLC analyses through fraction collection and subsequent gamma analysis (Na(Tl)) of the astatinated Trastuzumab (Activity) compared to UV absorption of the neat antibody (UV) as well as fraction collected activity of the product after > 24 h (Activity day 2). Raw data in Table ST8 and ST9.

Table ST11. FPLC fraction collected activity data

At-211 Day 1 (CPM)	At-211 Day 2 (CPM)	fractions (1/min)
0	28,87	1
0	0,95	2
8,93	8,88	3
0,95	0	4
0	0	5
4,94	12,92	6
0	0,51	7
31,54	23,55	8
4,94	8,87	9
0,95	0	10
0	8,93	11
0,51	0,95	12
0	0	13
104,07	8,93	14
116,63	20,89	15
535,51	264,24	16
5491,37	3131,97	17
42531,07	21502,66	18
82455,27	57035,1	19

35916,76	37900,13	20
23740,03	36616,41	21
598924,54	530721,43	22
1583085,4	1179834,62	23
765042,16	669213,55	24
283231,1	403242,85	25
109207,4	160420,37	26
45904,03	61025,87	27
24921,69	41267,44	28
17008,75	37711,68	29
25533,68	117068,02	30
31273,91	156606,41	31
19870,25	81283,05	32
11207,2	37448,34	33
6701,63	20837,76	34
4574,34	13084,14	35
3798,37	9369,14	36
3288,04	7996,27	37
2653,61	7969,67	38
2719,67	8430,54	39
2471,84	7173,96	40
2374,31	5390,27	41
2031,16	5494,47	42
1420,29	4415,16	43
1203,96	3383,72	44
1173,38	3296,03	45
997,39	2552,08	46
901,2	2418,19	47
836,93	2630,1	48
809,89	2062,34	49
674,37	1719,52	50
962,45	2049,93	51
689,76	2417,75	52
519,55	2178,36	53
582,72	1764,6	54
579,39	1399,01	55
554,38	1344,93	56
2808,34	1500,09	57
647,21	1549,81	58
491,63	2245,73	59
447,3	4365,48	60
462,37	3184,32	61
435,77	1929,32	62
423,36	1221,25	63
435,78	958,39	64
443,75	746,06	65

427,35	773,54	66
415,39	1009,81	67
455,72	2120,63	68
623,27	5113,55	69
674,69	5134,38	70
379,93	1770,94	71

Table ST12. FPLC UV data

Time (min)	UV (280 nm)	Time (min)	UV (280 nm)	Time (min)	UV (280 nm)	Time (min)	UV (280 nm)	Time (min)	UV (280 nm)	Time (min)	UV (280 nm)
0	0,004	12	0,131	24	5,536	36	-0,101	48	-0,12	60	-0,132
0,04	-0,001	12,04	0,128	24,04	5,533	36,04	-0,111	48,04	-0,123	60,04	-0,143
0,08	-0,006	12,08	0,135	24,08	5,52	36,08	-0,108	48,08	-0,112	60,08	-0,152
0,12	0,027	12,12	0,135	24,12	5,498	36,12	-0,091	48,12	-0,107	60,12	-0,136
0,16	0,1	12,16	0,134	24,16	5,469	36,16	-0,088	48,16	-0,115	60,16	-0,133
0,2	0,144	12,2	0,146	24,2	5,42	36,2	-0,09	48,2	-0,116	60,2	-0,138
0,24	0,138	12,24	0,155	24,24	5,363	36,24	-0,096	48,24	-0,118	60,24	-0,132
0,28	0,129	12,28	0,157	24,28	5,285	36,28	-0,093	48,28	-0,121	60,28	-0,131
0,32	0,131	12,32	0,162	24,32	5,162	36,32	-0,091	48,32	-0,119	60,32	-0,134
0,36	0,12	12,36	0,175	24,36	5,025	36,36	-0,098	48,36	-0,119	60,36	-0,145
0,4	0,119	12,4	0,181	24,4	4,869	36,4	-0,102	48,4	-0,112	60,4	-0,148
0,44	0,123	12,44	0,193	24,44	4,681	36,44	-0,1	48,44	-0,113	60,44	-0,145
0,48	0,109	12,48	0,224	24,48	4,474	36,48	-0,107	48,48	-0,113	60,48	-0,131
0,52	0,095	12,52	0,228	24,52	4,243	36,52	-0,103	48,52	-0,107	60,52	-0,133
0,56	0,095	12,56	0,238	24,56	4,027	36,56	-0,09	48,56	-0,11	60,56	-0,139
0,6	0,107	12,6	0,251	24,6	3,806	36,6	-0,087	48,6	-0,109	60,6	-0,144
0,64	0,105	12,64	0,273	24,64	3,579	36,64	-0,093	48,64	-0,116	60,64	-0,141
0,68	0,091	12,68	0,281	24,68	3,359	36,68	-0,094	48,68	-0,118	60,68	-0,131
0,72	0,095	12,72	0,296	24,72	3,139	36,72	-0,093	48,72	-0,111	60,72	-0,132
0,76	0,087	12,76	0,301	24,76	2,927	36,76	-0,085	48,76	-0,121	60,76	-0,132
0,8	0,084	12,8	0,293	24,8	2,719	36,8	-0,082	48,8	-0,116	60,8	-0,133
0,84	0,082	12,84	0,292	24,84	2,524	36,84	-0,081	48,84	-0,117	60,84	-0,133
0,88	0,073	12,88	0,298	24,88	2,341	36,88	-0,085	48,88	-0,133	60,88	-0,131
0,92	0,069	12,92	0,308	24,92	2,175	36,92	-0,079	48,92	-0,127	60,92	-0,146
0,96	0,061	12,96	0,3	24,96	2,026	36,96	-0,088	48,96	-0,121	60,96	-0,15
1	0,063	13	0,309	25	1,893	37	-0,099	49	-0,123	61	-0,151
1,04	0,048	13,04	0,308	25,04	1,75	37,04	-0,094	49,04	-0,113	61,04	-0,139
1,08	0,052	13,08	0,303	25,08	1,631	37,08	-0,089	49,08	-0,109	61,08	-0,139
1,12	0,057	13,12	0,301	25,12	1,53	37,12	-0,095	49,12	-0,116	61,12	-0,133
1,16	0,062	13,16	0,324	25,16	1,413	37,16	-0,099	49,16	-0,115	61,16	-0,141
1,2	0,091	13,2	0,339	25,2	1,321	37,2	-0,097	49,2	-0,1	61,2	-0,144
1,24	0,085	13,24	0,347	25,24	1,243	37,24	-0,098	49,24	-0,103	61,24	-0,134
1,28	0,08	13,28	0,346	25,28	1,17	37,28	-0,11	49,28	-0,115	61,28	-0,149
1,32	0,072	13,32	0,334	25,32	1,088	37,32	-0,105	49,32	-0,109	61,32	-0,154
1,36	0,062	13,36	0,343	25,36	1,024	37,36	-0,102	49,36	-0,102	61,36	-0,145
1,4	0,065	13,4	0,356	25,4	0,971	37,4	-0,104	49,4	-0,11	61,4	-0,146
1,44	0,076	13,44	0,368	25,44	0,907	37,44	-0,117	49,44	-0,118	61,44	-0,144
1,48	0,067	13,48	0,378	25,48	0,852	37,48	-0,115	49,48	-0,125	61,48	-0,144
1,52	0,056	13,52	0,383	25,52	0,808	37,52	-0,104	49,52	-0,133	61,52	-0,131
1,56	0,064	13,56	0,388	25,56	0,758	37,56	-0,102	49,56	-0,122	61,56	-0,128
1,6	0,056	13,6	0,398	25,6	0,715	37,6	-0,101	49,6	-0,115	61,6	-0,137
1,64	0,056	13,64	0,397	25,64	0,679	37,64	-0,098	49,64	-0,123	61,64	-0,144
1,68	0,053	13,68	0,398	25,68	0,644	37,68	-0,111	49,68	-0,122	61,68	-0,147

1,72	0,038	13,72	0,401	25,72	0,599	37,72	-0,115	49,72	-0,119	61,72	-0,143
1,76	0,027	13,76	0,397	25,76	0,56	37,76	-0,117	49,76	-0,104	61,76	-0,138
1,8	0,032	13,8	0,414	25,8	0,532	37,8	-0,123	49,8	-0,103	61,8	-0,138
1,84	0,036	13,84	0,412	25,84	0,505	37,84	-0,117	49,84	-0,113	61,84	-0,134
1,88	0,034	13,88	0,411	25,88	0,475	37,88	-0,109	49,88	-0,112	61,88	-0,124
1,92	0,022	13,92	0,42	25,92	0,446	37,92	-0,12	49,92	-0,118	61,92	-0,127
1,96	0,017	13,96	0,408	25,96	0,406	37,96	-0,129	49,96	-0,117	61,96	-0,133
2	0,024	14	0,409	26	0,374	38	-0,125	50	-0,121	62	-0,131
2,04	-0,098	14,04	0,412	26,04	0,343	38,04	-0,12	50,04	-0,123	62,04	-0,125
2,08	-0,07	14,08	0,414	26,08	0,335	38,08	-0,121	50,08	-0,13	62,08	-0,135
2,12	-0,005	14,12	0,41	26,12	0,328	38,12	-0,131	50,12	-0,125	62,12	-0,14
2,16	0,648	14,16	0,406	26,16	0,301	38,16	-0,139	50,16	-0,115	62,16	-0,129
2,2	5,715	14,2	0,424	26,2	0,287	38,2	-0,151	50,2	-0,104	62,2	-0,128
2,24	12,539	14,24	0,429	26,24	0,283	38,24	-0,151	50,24	-0,116	62,24	-0,125
2,28	14,698	14,28	0,426	26,28	0,258	38,28	-0,151	50,28	-0,121	62,28	-0,126
2,32	12,893	14,32	0,422	26,32	0,234	38,32	-0,149	50,32	-0,114	62,32	-0,123
2,36	11,55	14,36	0,426	26,36	0,221	38,36	-0,153	50,36	-0,118	62,36	-0,128
2,4	11,039	14,4	0,417	26,4	0,214	38,4	-0,163	50,4	-0,115	62,4	-0,135
2,44	10,779	14,44	0,418	26,44	0,21	38,44	-0,167	50,44	-0,116	62,44	-0,134
2,48	10,564	14,48	0,429	26,48	0,188	38,48	-0,164	50,48	-0,12	62,48	-0,123
2,52	10,288	14,52	0,435	26,52	0,176	38,52	-0,154	50,52	-0,123	62,52	-0,116
2,56	9,973	14,56	0,445	26,56	0,174	38,56	-0,159	50,56	-0,125	62,56	-0,121
2,6	9,594	14,6	0,451	26,6	0,173	38,6	-0,175	50,6	-0,129	62,6	-0,117
2,64	9,175	14,64	0,448	26,64	0,162	38,64	-0,171	50,64	-0,109	62,64	-0,106
2,68	8,797	14,68	0,436	26,68	0,157	38,68	-0,173	50,68	-0,109	62,68	-0,108
2,72	8,409	14,72	0,423	26,72	0,151	38,72	-0,166	50,72	-0,11	62,72	-0,117
2,76	7,994	14,76	0,43	26,76	0,14	38,76	-0,17	50,76	-0,107	62,76	-0,125
2,8	7,59	14,8	0,435	26,8	0,137	38,8	-0,162	50,8	-0,111	62,8	-0,121
2,84	7,237	14,84	0,436	26,84	0,123	38,84	-0,154	50,84	-0,116	62,84	-0,123
2,88	6,884	14,88	0,43	26,88	0,104	38,88	-0,152	50,88	-0,112	62,88	-0,113
2,92	6,516	14,92	0,418	26,92	0,096	38,92	-0,163	50,92	-0,117	62,92	-0,101
2,96	6,19	14,96	0,407	26,96	0,097	38,96	-0,16	50,96	-0,13	62,96	-0,101
3	5,885	15	0,411	27	0,099	39	-0,161	51	-0,126	63	-0,112
3,04	5,598	15,04	0,409	27,04	0,099	39,04	-0,162	51,04	-0,12	63,04	-0,121
3,08	5,322	15,08	0,4	27,08	0,088	39,08	-0,164	51,08	-0,114	63,08	-0,116
3,12	5,036	15,12	0,388	27,12	0,075	39,12	-0,165	51,12	-0,12	63,12	-0,117
3,16	4,781	15,16	0,382	27,16	0,073	39,16	-0,163	51,16	-0,126	63,16	-0,117
3,2	4,549	15,2	0,376	27,2	0,082	39,2	-0,166	51,2	-0,131	63,2	-0,123
3,24	4,338	15,24	0,357	27,24	0,084	39,24	-0,168	51,24	-0,124	63,24	-0,129
3,28	4,119	15,28	0,348	27,28	0,073	39,28	-0,16	51,28	-0,12	63,28	-0,122
3,32	3,909	15,32	0,341	27,32	0,073	39,32	-0,165	51,32	-0,129	63,32	-0,119
3,36	3,712	15,36	0,339	27,36	0,076	39,36	-0,165	51,36	-0,133	63,36	-0,107
3,4	3,532	15,4	0,332	27,4	0,068	39,4	-0,162	51,4	-0,12	63,4	-0,121
3,44	3,381	15,44	0,337	27,44	0,06	39,44	-0,17	51,44	-0,113	63,44	-0,138
3,48	3,236	15,48	0,334	27,48	0,071	39,48	-0,164	51,48	-0,119	63,48	-0,137
3,52	3,079	15,52	0,322	27,52	0,072	39,52	-0,156	51,52	-0,125	63,52	-0,129

3,56	2,936	15,56	0,307	27,56	0,062	39,56	-0,151	51,56	-0,128	63,56	-0,137
3,6	2,812	15,6	0,299	27,6	0,063	39,6	-0,145	51,6	-0,116	63,6	-0,146
3,64	2,683	15,64	0,309	27,64	0,068	39,64	-0,147	51,64	-0,113	63,64	-0,138
3,68	2,577	15,68	0,311	27,68	0,07	39,68	-0,139	51,68	-0,115	63,68	-0,138
3,72	2,476	15,72	0,308	27,72	0,054	39,72	-0,121	51,72	-0,113	63,72	-0,149
3,76	2,363	15,76	0,304	27,76	0,055	39,76	-0,118	51,76	-0,107	63,76	-0,156
3,8	2,272	15,8	0,289	27,8	0,061	39,8	-0,11	51,8	-0,118	63,8	-0,156
3,84	2,184	15,84	0,276	27,84	0,062	39,84	-0,102	51,84	-0,121	63,84	-0,145
3,88	2,105	15,88	0,27	27,88	0,067	39,88	-0,098	51,88	-0,129	63,88	-0,14
3,92	2,025	15,92	0,268	27,92	0,062	39,92	-0,096	51,92	-0,128	63,92	-0,149
3,96	1,938	15,96	0,254	27,96	0,055	39,96	-0,094	51,96	-0,119	63,96	-0,157
4	1,867	16	0,24	28	0,051	40	-0,101	52	-0,111	64	-0,169
4,04	1,801	16,04	0,246	28,04	0,064	40,04	-0,084	52,04	-0,114	64,04	-0,176
4,08	1,743	16,08	0,251	28,08	0,073	40,08	-0,076	52,08	-0,123	64,08	-0,171
4,12	1,675	16,12	0,254	28,12	0,066	40,12	-0,079	52,12	-0,12	64,12	-0,164
4,16	1,618	16,16	0,247	28,16	0,054	40,16	-0,086	52,16	-0,129	64,16	-0,156
4,2	1,567	16,2	0,244	28,2	0,05	40,2	-0,086	52,2	-0,132	64,2	-0,156
4,24	1,523	16,24	0,23	28,24	0,05	40,24	-0,089	52,24	-0,122	64,24	-0,164
4,28	1,487	16,28	0,215	28,28	0,041	40,28	-0,094	52,28	-0,118	64,28	-0,164
4,32	1,446	16,32	0,217	28,32	0,025	40,32	-0,096	52,32	-0,123	64,32	-0,17
4,36	1,402	16,36	0,207	28,36	0,039	40,36	-0,097	52,36	-0,122	64,36	-0,16
4,4	1,361	16,4	0,215	28,4	0,047	40,4	-0,084	52,4	-0,121	64,4	-0,15
4,44	1,312	16,44	0,213	28,44	0,041	40,44	-0,091	52,44	-0,128	64,44	-0,154
4,48	1,272	16,48	0,22	28,48	0,026	40,48	-0,096	52,48	-0,143	64,48	-0,168
4,52	1,248	16,52	0,208	28,52	0,023	40,52	-0,097	52,52	-0,136	64,52	-0,182
4,56	1,21	16,56	0,213	28,56	0,034	40,56	-0,096	52,56	-0,132	64,56	-0,176
4,6	1,16	16,6	0,215	28,6	0,038	40,6	-0,094	52,6	-0,138	64,6	-0,161
4,64	1,124	16,64	0,204	28,64	0,036	40,64	-0,097	52,64	-0,139	64,64	-0,164
4,68	1,098	16,68	0,211	28,68	0,038	40,68	-0,111	52,68	-0,14	64,68	-0,17
4,72	1,084	16,72	0,209	28,72	0,043	40,72	-0,129	52,72	-0,143	64,72	-0,17
4,76	1,046	16,76	0,216	28,76	0,039	40,76	-0,129	52,76	-0,148	64,76	-0,175
4,8	0,997	16,8	0,216	28,8	0,043	40,8	-0,128	52,8	-0,149	64,8	-0,177
4,84	0,97	16,84	0,221	28,84	0,054	40,84	-0,127	52,84	-0,142	64,84	-0,179
4,88	0,953	16,88	0,216	28,88	0,066	40,88	-0,131	52,88	-0,148	64,88	-0,18
4,92	0,94	16,92	0,211	28,92	0,075	40,92	-0,139	52,92	-0,143	64,92	-0,184
4,96	0,907	16,96	0,219	28,96	0,071	40,96	-0,149	52,96	-0,142	64,96	-0,19
5	0,896	17	0,216	29	0,082	41	-0,163	53	-0,153	65	-0,187
5,04	0,886	17,04	0,213	29,04	0,108	41,04	-0,163	53,04	-0,164	65,04	-0,178
5,08	0,86	17,08	0,208	29,08	0,121	41,08	-0,178	53,08	-0,164	65,08	-0,184
5,12	0,82	17,12	0,207	29,12	0,142	41,12	-0,185	53,12	-0,164	65,12	-0,189
5,16	0,813	17,16	0,209	29,16	0,171	41,16	-0,184	53,16	-0,155	65,16	-0,188
5,2	0,81	17,2	0,212	29,2	0,195	41,2	-0,181	53,2	-0,154	65,2	-0,197
5,24	0,796	17,24	0,214	29,24	0,218	41,24	-0,181	53,24	-0,16	65,24	-0,196
5,28	0,769	17,28	0,206	29,28	0,254	41,28	-0,196	53,28	-0,151	65,28	-0,187
5,32	0,737	17,32	0,212	29,32	0,296	41,32	-0,207	53,32	-0,158	65,32	-0,183
5,36	0,704	17,36	0,208	29,36	0,357	41,36	-0,208	53,36	-0,155	65,36	-0,173

5,4	0,699	17,4	0,222	29,4	0,417	41,4	-0,202	53,4	-0,15	65,4	-0,171
5,44	0,696	17,44	0,238	29,44	0,474	41,44	-0,203	53,44	-0,163	65,44	-0,177
5,48	0,685	17,48	0,249	29,48	0,522	41,48	-0,2	53,48	-0,166	65,48	-0,175
5,52	0,682	17,52	0,272	29,52	0,598	41,52	-0,198	53,52	-0,166	65,52	-0,17
5,56	0,67	17,56	0,301	29,56	0,681	41,56	-0,193	53,56	-0,156	65,56	-0,17
5,6	0,65	17,6	0,34	29,6	0,745	41,6	-0,191	53,6	-0,152	65,6	-0,173
5,64	0,647	17,64	0,382	29,64	0,805	41,64	-0,194	53,64	-0,152	65,64	-0,179
5,68	0,635	17,68	0,433	29,68	0,885	41,68	-0,202	53,68	-0,14	65,68	-0,18
5,72	0,628	17,72	0,502	29,72	0,958	41,72	-0,195	53,72	-0,149	65,72	-0,186
5,76	0,623	17,76	0,568	29,76	1,016	41,76	-0,179	53,76	-0,149	65,76	-0,182
5,8	0,604	17,8	0,63	29,8	1,082	41,8	-0,177	53,8	-0,147	65,8	-0,18
5,84	0,582	17,84	0,699	29,84	1,142	41,84	-0,167	53,84	-0,15	65,84	-0,181
5,88	0,574	17,88	0,788	29,88	1,169	41,88	-0,154	53,88	-0,151	65,88	-0,18
5,92	0,573	17,92	0,864	29,92	1,19	41,92	-0,152	53,92	-0,14	65,92	-0,179
5,96	0,558	17,96	0,934	29,96	1,201	41,96	-0,152	53,96	-0,134	65,96	-0,178
6	0,542	18	1,006	30	1,198	42	-0,142	54	-0,136	66	-0,17
6,04	0,548	18,04	1,067	30,04	1,202	42,04	-0,149	54,04	-0,143	66,04	-0,176
6,08	0,539	18,08	1,121	30,08	1,191	42,08	-0,14	54,08	-0,137	66,08	-0,187
6,12	0,52	18,12	1,16	30,12	1,166	42,12	-0,145	54,12	-0,138	66,12	-0,182
6,16	0,525	18,16	1,211	30,16	1,138	42,16	-0,145	54,16	-0,147	66,16	-0,192
6,2	0,508	18,2	1,253	30,2	1,098	42,2	-0,151	54,2	-0,138	66,2	-0,191
6,24	0,508	18,24	1,286	30,24	1,044	42,24	-0,149	54,24	-0,134	66,24	-0,197
6,28	0,501	18,28	1,319	30,28	0,991	42,28	-0,13	54,28	-0,132	66,28	-0,189
6,32	0,493	18,32	1,339	30,32	0,933	42,32	-0,136	54,32	-0,137	66,32	-0,185
6,36	0,486	18,36	1,352	30,36	0,87	42,36	-0,136	54,36	-0,143	66,36	-0,182
6,4	0,458	18,4	1,372	30,4	0,815	42,4	-0,124	54,4	-0,145	66,4	-0,173
6,44	0,456	18,44	1,38	30,44	0,755	42,44	-0,112	54,44	-0,138	66,44	-0,173
6,48	0,452	18,48	1,379	30,48	0,697	42,48	-0,117	54,48	-0,14	66,48	-0,183
6,52	0,448	18,52	1,373	30,52	0,645	42,52	-0,111	54,52	-0,134	66,52	-0,189
6,56	0,451	18,56	1,366	30,56	0,589	42,56	-0,112	54,56	-0,129	66,56	-0,179
6,6	0,441	18,6	1,349	30,6	0,542	42,6	-0,111	54,6	-0,128	66,6	-0,184
6,64	0,43	18,64	1,323	30,64	0,481	42,64	-0,107	54,64	-0,121	66,64	-0,191
6,68	0,435	18,68	1,293	30,68	0,436	42,68	-0,116	54,68	-0,123	66,68	-0,194
6,72	0,42	18,72	1,273	30,72	0,399	42,72	-0,116	54,72	-0,129	66,72	-0,18
6,76	0,414	18,76	1,252	30,76	0,355	42,76	-0,11	54,76	-0,141	66,76	-0,182
6,8	0,42	18,8	1,211	30,8	0,314	42,8	-0,102	54,8	-0,146	66,8	-0,178
6,84	0,415	18,84	1,157	30,84	0,282	42,84	-0,101	54,84	-0,145	66,84	-0,17
6,88	0,398	18,88	1,102	30,88	0,258	42,88	-0,117	54,88	-0,144	66,88	-0,187
6,92	0,394	18,92	1,048	30,92	0,229	42,92	-0,107	54,92	-0,144	66,92	-0,193
6,96	0,395	18,96	1	30,96	0,201	42,96	-0,106	54,96	-0,127	66,96	-0,183
7	0,393	19	0,966	31	0,171	43	-0,112	55	-0,128	67	-0,174
7,04	0,392	19,04	0,915	31,04	0,138	43,04	-0,1	55,04	-0,14	67,04	-0,175
7,08	0,389	19,08	0,857	31,08	0,118	43,08	-0,1	55,08	-0,129	67,08	-0,171
7,12	0,392	19,12	0,8	31,12	0,111	43,12	-0,114	55,12	-0,12	67,12	-0,182
7,16	0,386	19,16	0,752	31,16	0,095	43,16	-0,112	55,16	-0,126	67,16	-0,191
7,2	0,369	19,2	0,718	31,2	0,068	43,2	-0,109	55,2	-0,131	67,2	-0,178

7,24	0,362	19,24	0,677	31,24	0,059	43,24	-0,109	55,24	-0,141	67,24	-0,18
7,28	0,366	19,28	0,627	31,28	0,056	43,28	-0,105	55,28	-0,142	67,28	-0,192
7,32	0,365	19,32	0,586	31,32	0,039	43,32	-0,103	55,32	-0,139	67,32	-0,192
7,36	0,351	19,36	0,557	31,36	0,035	43,36	-0,092	55,36	-0,131	67,36	-0,197
7,4	0,357	19,4	0,532	31,4	0,023	43,4	-0,101	55,4	-0,133	67,4	-0,189
7,44	0,341	19,44	0,505	31,44	0,022	43,44	-0,105	55,44	-0,131	67,44	-0,186
7,48	0,328	19,48	0,466	31,48	0,024	43,48	-0,09	55,48	-0,133	67,48	-0,192
7,52	0,328	19,52	0,43	31,52	0,008	43,52	-0,096	55,52	-0,131	67,52	-0,182
7,56	0,321	19,56	0,42	31,56	0	43,56	-0,097	55,56	-0,14	67,56	-0,18
7,6	0,32	19,6	0,397	31,6	0	43,6	-0,097	55,6	-0,13	67,6	-0,183
7,64	0,319	19,64	0,375	31,64	-0,003	43,64	-0,096	55,64	-0,123	67,64	-0,192
7,68	0,316	19,68	0,364	31,68	-0,001	43,68	-0,092	55,68	-0,121	67,68	-0,187
7,72	0,314	19,72	0,353	31,72	-0,003	43,72	-0,093	55,72	-0,119	67,72	-0,19
7,76	0,313	19,76	0,351	31,76	-0,011	43,76	-0,096	55,76	-0,121	67,76	-0,2
7,8	0,302	19,8	0,344	31,8	-0,007	43,8	-0,095	55,8	-0,135	67,8	-0,21
7,84	0,296	19,84	0,344	31,84	-0,003	43,84	-0,083	55,84	-0,138	67,84	-0,2
7,88	0,296	19,88	0,349	31,88	-0,003	43,88	-0,069	55,88	-0,128	67,88	-0,181
7,92	0,289	19,92	0,359	31,92	-0,012	43,92	-0,072	55,92	-0,136	67,92	-0,186
7,96	0,276	19,96	0,363	31,96	-0,029	43,96	-0,063	55,96	-0,136	67,96	-0,194
8	0,283	20	0,365	32	-0,028	44	-0,07	56	-0,125	68	-0,202
8,04	0,283	20,04	0,368	32,04	-0,024	44,04	-0,066	56,04	-0,119	68,04	-0,191
8,08	0,274	20,08	0,38	32,08	-0,037	44,08	-0,067	56,08	-0,127	68,08	-0,191
8,12	0,273	20,12	0,394	32,12	-0,041	44,12	-0,067	56,12	-0,139	68,12	-0,19
8,16	0,275	20,16	0,4	32,16	-0,042	44,16	-0,056	56,16	-0,136	68,16	-0,188
8,2	0,277	20,2	0,405	32,2	-0,036	44,2	-0,043	56,2	-0,13	68,2	-0,194
8,24	0,274	20,24	0,416	32,24	-0,04	44,24	-0,045	56,24	-0,131	68,24	-0,189
8,28	0,279	20,28	0,438	32,28	-0,058	44,28	-0,04	56,28	-0,127	68,28	-0,187
8,32	0,27	20,32	0,435	32,32	-0,044	44,32	-0,04	56,32	-0,126	68,32	-0,188
8,36	0,262	20,36	0,434	32,36	-0,042	44,36	-0,047	56,36	-0,134	68,36	-0,193
8,4	0,263	20,4	0,449	32,4	-0,042	44,4	-0,039	56,4	-0,131	68,4	-0,197
8,44	0,255	20,44	0,449	32,44	-0,039	44,44	-0,043	56,44	-0,126	68,44	-0,189
8,48	0,258	20,48	0,457	32,48	-0,048	44,48	-0,051	56,48	-0,124	68,48	-0,18
8,52	0,261	20,52	0,475	32,52	-0,063	44,52	-0,055	56,52	-0,142	68,52	-0,179
8,56	0,245	20,56	0,496	32,56	-0,064	44,56	-0,054	56,56	-0,154	68,56	-0,191
8,6	0,232	20,6	0,499	32,6	-0,057	44,6	-0,049	56,6	-0,14	68,6	-0,196
8,64	0,226	20,64	0,509	32,64	-0,057	44,64	-0,048	56,64	-0,128	68,64	-0,184
8,68	0,229	20,68	0,53	32,68	-0,059	44,68	-0,046	56,68	-0,13	68,68	-0,195
8,72	0,233	20,72	0,554	32,72	-0,055	44,72	-0,043	56,72	-0,13	68,72	-0,192
8,76	0,234	20,76	0,568	32,76	-0,066	44,76	-0,044	56,76	-0,128	68,76	-0,187
8,8	0,233	20,8	0,588	32,8	-0,07	44,8	-0,037	56,8	-0,128	68,8	-0,211
8,84	0,229	20,84	0,619	32,84	-0,057	44,84	-0,044	56,84	-0,124	68,84	-0,211
8,88	0,225	20,88	0,665	32,88	-0,054	44,88	-0,047	56,88	-0,13	68,88	-0,202
8,92	0,246	20,92	0,722	32,92	-0,069	44,92	-0,053	56,92	-0,128	68,92	-0,201
8,96	0,244	20,96	0,778	32,96	-0,079	44,96	-0,051	56,96	-0,129	68,96	-0,191
9	0,224	21	0,851	33	-0,07	45	-0,065	57	-0,119	69	-0,189
9,04	0,22	21,04	0,931	33,04	-0,062	45,04	-0,081	57,04	-0,117	69,04	-0,201

9,08	0,226	21,08	1,021	33,08	-0,065	45,08	-0,084	57,08	-0,115	69,08	-0,196
9,12	0,232	21,12	1,147	33,12	-0,074	45,12	-0,081	57,12	-0,106	69,12	-0,191
9,16	0,236	21,16	1,329	33,16	-0,071	45,16	-0,085	57,16	-0,115	69,16	-0,192
9,2	0,223	21,2	1,55	33,2	-0,073	45,2	-0,083	57,2	-0,112	69,2	-0,191
9,24	0,209	21,24	1,845	33,24	-0,08	45,24	-0,073	57,24	-0,113	69,24	-0,195
9,28	0,219	21,28	2,225	33,28	-0,072	45,28	-0,074	57,28	-0,122	69,28	-0,195
9,32	0,217	21,32	2,716	33,32	-0,065	45,32	-0,085	57,32	-0,105	69,32	-0,194
9,36	0,206	21,36	3,372	33,36	-0,064	45,36	-0,089	57,36	-0,114	69,36	-0,194
9,4	0,206	21,4	4,246	33,4	-0,072	45,4	-0,09	57,4	-0,115	69,4	-0,196
9,44	0,199	21,44	5,371	33,44	-0,078	45,44	-0,086	57,44	-0,116	69,44	-0,201
9,48	0,187	21,48	6,786	33,48	-0,065	45,48	-0,092	57,48	-0,127	69,48	-0,199
9,52	0,185	21,52	8,626	33,52	-0,059	45,52	-0,103	57,52	-0,121	69,52	-0,192
9,56	0,188	21,56	10,931	33,56	-0,076	45,56	-0,106	57,56	-0,124	69,56	-0,19
9,6	0,192	21,6	13,775	33,6	-0,082	45,6	-0,1	57,6	-0,122	69,6	-0,182
9,64	0,195	21,64	17,171	33,64	-0,085	45,64	-0,096	57,64	-0,124	69,64	-0,188
9,68	0,197	21,68	21,232	33,68	-0,079	45,68	-0,095	57,68	-0,118	69,68	-0,195
9,72	0,18	21,72	25,925	33,72	-0,071	45,72	-0,1	57,72	-0,117	69,72	-0,19
9,76	0,176	21,76	31,222	33,76	-0,073	45,76	-0,098	57,76	-0,111	69,76	-0,186
9,8	0,174	21,8	37,197	33,8	-0,074	45,8	-0,09	57,8	-0,121	69,8	-0,189
9,84	0,185	21,84	43,686	33,84	-0,068	45,84	-0,095	57,84	-0,125	69,84	-0,191
9,88	0,177	21,88	50,534	33,88	-0,072	45,88	-0,099	57,88	-0,115	69,88	-0,19
9,92	0,176	21,92	57,618	33,92	-0,083	45,92	-0,107	57,92	-0,119	69,92	-0,182
9,96	0,173	21,96	64,856	33,96	-0,08	45,96	-0,1	57,96	-0,124	69,96	-0,19
10	0,163	22	71,872	34	-0,071	46	-0,092	58	-0,127	70	-0,209
10,04	0,157	22,04	78,444	34,04	-0,074	46,04	-0,101	58,04	-0,13	70,04	-0,202
10,08	0,159	22,08	84,541	34,08	-0,063	46,08	-0,101	58,08	-0,129	70,08	-0,193
10,12	0,17	22,12	89,921	34,12	-0,052	46,12	-0,099	58,12	-0,113	70,12	-0,193
10,16	0,167	22,16	94,261	34,16	-0,071	46,16	-0,093	58,16	-0,105	70,16	-0,197
10,2	0,172	22,2	97,495	34,2	-0,081	46,2	-0,097	58,2	-0,112	70,2	-0,196
10,24	0,163	22,24	99,814	34,24	-0,071	46,24	-0,096	58,24	-0,117	70,24	-0,195
10,28	0,151	22,28	100,887	34,28	-0,084	46,28	-0,096	58,28	-0,116	70,28	-0,191
10,32	0,16	22,32	100,766	34,32	-0,087	46,32	-0,1	58,32	-0,119	70,32	-0,192
10,36	0,155	22,36	99,641	34,36	-0,074	46,36	-0,098	58,36	-0,126	70,36	-0,199
10,4	0,145	22,4	97,636	34,4	-0,07	46,4	-0,108	58,4	-0,121	70,4	-0,202
10,44	0,14	22,44	94,738	34,44	-0,081	46,44	-0,106	58,44	-0,127	70,44	-0,2
10,48	0,148	22,48	91,107	34,48	-0,081	46,48	-0,092	58,48	-0,145	70,48	-0,209
10,52	0,149	22,52	87,081	34,52	-0,076	46,52	-0,094	58,52	-0,141	70,52	-0,21
10,56	0,153	22,56	82,613	34,56	-0,084	46,56	-0,09	58,56	-0,139	70,56	-0,195
10,6	0,16	22,6	77,868	34,6	-0,079	46,6	-0,097	58,6	-0,135	70,6	-0,203
10,64	0,15	22,64	72,959	34,64	-0,083	46,64	-0,102	58,64	-0,138	70,64	-0,2
10,68	0,144	22,68	68,1	34,68	-0,082	46,68	-0,102	58,68	-0,139	70,68	-0,194
10,72	0,148	22,72	63,283	34,72	-0,081	46,72	-0,107	58,72	-0,129	70,72	-0,216
10,76	0,148	22,76	58,541	34,76	-0,082	46,76	-0,106	58,76	-0,13	70,76	-0,221
10,8	0,137	22,8	54,026	34,8	-0,08	46,8	-0,104	58,8	-0,132	70,8	-0,201
10,84	0,127	22,84	49,673	34,84	-0,077	46,84	-0,106	58,84	-0,127	70,84	-0,206
10,88	0,128	22,88	45,529	34,88	-0,079	46,88	-0,108	58,88	-0,13	70,88	-0,207

10,92	0,125	22,92	41,615	34,92	-0,066	46,92	-0,102	58,92	-0,141	70,92	-0,214
10,96	0,125	22,96	37,977	34,96	-0,058	46,96	-0,103	58,96	-0,134	70,96	-0,212
11	0,127	23	34,564	35	-0,071	47	-0,105	59	-0,137	71	-0,204
11,04	0,128	23,04	31,375	35,04	-0,076	47,04	-0,111	59,04	-0,14	71,04	-0,209
11,08	0,141	23,08	28,45	35,08	-0,072	47,08	-0,112	59,08	-0,141	71,08	-0,223
11,12	0,143	23,12	25,753	35,12	-0,071	47,12	-0,109	59,12	-0,137	71,12	-0,221
11,16	0,136	23,16	23,288	35,16	-0,078	47,16	-0,115	59,16	-0,138	71,16	-0,204
11,2	0,127	23,2	21,027	35,2	-0,084	47,2	-0,121	59,2	-0,154	71,2	-0,202
11,24	0,127	23,24	18,957	35,24	-0,084	47,24	-0,117	59,24	-0,149	71,24	-0,211
11,28	0,14	23,28	17,103	35,28	-0,096	47,28	-0,116	59,28	-0,145	71,28	-0,216
11,32	0,136	23,32	15,444	35,32	-0,089	47,32	-0,12	59,32	-0,148	71,32	-0,214
11,36	0,14	23,36	13,959	35,36	-0,083	47,36	-0,11	59,36	-0,143	71,36	-0,215
11,4	0,135	23,4	12,648	35,4	-0,089	47,4	-0,113	59,4	-0,155	71,4	-0,211
11,44	0,125	23,44	11,492	35,44	-0,078	47,44	-0,124	59,44	-0,161	71,44	-0,209
11,48	0,123	23,48	10,449	35,48	-0,072	47,48	-0,132	59,48	-0,149	71,48	-0,214
11,52	0,13	23,52	9,535	35,52	-0,082	47,52	-0,116	59,52	-0,137	71,52	-0,201
11,56	0,136	23,56	8,745	35,56	-0,092	47,56	-0,108	59,56	-0,135	71,56	-0,2
11,6	0,143	23,6	8,081	35,6	-0,096	47,6	-0,119	59,6	-0,134	71,6	-0,208
11,64	0,141	23,64	7,495	35,64	-0,092	47,64	-0,138	59,64	-0,134	71,64	-0,211
11,68	0,131	23,68	7,009	35,68	-0,1	47,68	-0,141	59,68	-0,14	71,68	-0,205
11,72	0,129	23,72	6,621	35,72	-0,1	47,72	-0,141	59,72	-0,145	71,72	-0,212
11,76	0,123	23,76	6,303	35,76	-0,101	47,76	-0,135	59,76	-0,146	71,76	-0,214
11,8	0,134	23,8	6,04	35,8	-0,09	47,8	-0,128	59,8	-0,148	71,72	-0,212
11,84	0,142	23,84	5,841	35,84	-0,087	47,84	-0,128	59,84	-0,144	71,76	-0,214
11,88	0,126	23,88	5,712	35,88	-0,096	47,88	-0,127	59,88	-0,145		
11,92	0,131	23,92	5,626	35,92	-0,091	47,92	-0,127	59,92	-0,146		
11,96	0,131	23,96	5,563	35,96	-0,092	47,96	-0,12	59,96	-0,138		

Method validation for in-house QC methods:

The in-house QC methods of methanol precipitation, binding to HER2 substituted magnetic beads and FPLC analysis are validated according to performance repeatability, robustness and specificity.

Methanol precipitation use the actual product At-211-Trastuzumab for validation by repeated measurements to determine the radiochemical purity of the product and to ensure repeatability in calculated RCP. The dilution factor of the product is changed to ensure robustness of the method by maintaining the actual RCP. Finally, the product is doped with free astatine activity to show specificity by detecting a lower RCP.

Verifying the magnetic HER2 beads assay is conducted using directly radioiodinated Trastuzumab, using the iodogen method. Repeated assays show repeatability by comparing the B/T of Trastuzumab. Changing dilution factor of the beads is used to show robustness of the method by maintained B/T and the low binding of an iodinated unspecific antibody show specificity of the method.

FPLC similarly use directly radioiodinated Trastuzumab for verification. Repeatability is shown by repeated measurements of the verification product, including fraction collection, comparing the retention times. Robustness is shown by changing the mobile phase buffer while maintaining the same Rf. Doping the analyte with albumin in order to show simultaneous detection of different sized proteins show specificity of the method.
